# Prevalence and predictive factors associated with stunting in preschool children in a governorate of Iraq: a community-based cross-sectional study

**DOI:** 10.3389/fnut.2024.1322625

**Published:** 2024-02-14

**Authors:** Huda J. Mhamad, Zana B. Najmadden, Kaihan H. Hama Salih, Dlkhwaz A. Hama, Hiwa O. Abdullah, Karzan M. Hasan, Honar O. Kareem, Bilal A. Mohammed, Fattah H. Fattah, Berun A. Abdalla, Fahmi H. Kakamad, Shvan H. Mohammed

**Affiliations:** ^1^Food Science and Quality Control, Technical College of Applied Science, Sulaimani Polytechnic University, Sulaimani, Iraq; ^2^Research Center, University of Halabja, Halabja, Iraq; ^3^Smart Health Tower, Sulaimani, Iraq; ^4^Kscien Organization for Scientific Research, Sulaimani, Iraq; ^5^College of Medicine, University of Sulaimani, Sulaimani, Iraq

**Keywords:** child health care, growth retardation, malnutrition, nutritional status, stunting, survey

## Abstract

**Introduction:**

The prevalence and risk factors of stunting in various geographical regions have been well investigated. However, not enough data exists regarding the communities in Iraq. This study investigated the prevalence and risk factors of stunting in preschool children in Halabja governorate.

**Methods:**

The required data for the study was collected through a structured questionnaire form from the children’s parents. Then, the height and weight of the children were measured. According to the World Health Organization Child Growth Standards and using the WHO Anthro Survey Analyser software, children were classified as “stunted” when their height-for-age z-score was below two standard deviations.

**Results:**

A total of 646 children were included, of which 310 (48%) were male and 336 (52%) were female. The gestational age of 556 (86%) children was 9 months, while 84 (13%) were born between 7–9 months, and 6 (1%) were born in 7 months. Regarding feeding during the first 2 years of life, 229 children (35.4%) were exclusively breastfed, 93 (14.4%) were bottle-fed, and 324 (50.2%) had mixed feeding. The prevalence of stunting was 7.9% in the sample pool, with 4.6% of females and 3.3% of males. Among stunted children, 6.35% were term babies, and 1.55% were preterm babies. None of the studied factors had a significant association with stunting.

**Conclusion:**

The prevalence of stunting in the studied population was 7.9%. However, we could not find any significant association between the studied factors and stunting. Thus, the factors that may significantly affect stunting in our area of study, especially the historical chemical warfare side effects, need to be more extensively investigated in future studies.

## Introduction

The future prospects of infants and young children critically hinge on their optimal growth and development during the early stages of life. Stunting is a growth defect in which children have a lower height relative to their age and constitutes a serious health problem among children under the age of five in numerous low- and middle-income countries (1, 2). Stunting in children under the age of five can cause compromised physical development and may have an enduring effect on cognitive development, educational attainment, maternal reproductive outcomes, and economic productivity during adulthood (1, 2). As per United Nations estimates, one in eight Iraqi children dies before reaching 5 years of age. Nearly 30% experience malnutrition, a quarter are born underweight, and an additional 25% lack access to safe water (3).

The prevalence and risk factors of stunting in various geographical regions have been well investigated (1, 2, 4–6). However, not enough data exists regarding the communities in Iraq (3, 7, 8). The ongoing instability in Iraq since 2003 continues to have a profound impact on the physical and mental health of its residents (8). In a study conducted by Gabriela Guerrero-Serdan in Iraq to assess the effects of war on nutrition and health, it was hypothesized that those born after the war in high-intensity conflict areas were found to have lower height-for-age z-scores compared to those born in less violent areas (9).

This cross-sectional study explores the prevalence and risk factors of stunting in children aged four to five in the governorate of Halabja, which experienced chemical warfare in 1988.

## Method

### Study location and population

The study covered the urban areas in the Halabja Governorate of the Kurdistan Region, Iraq, with a total population of 121,793 people based on the Kurdistan Region Statistics Office.^1^ The study was specific to the kindergartens located inside the city.

### Study design

This was a survey-based cross-sectional study that enrolled kindergarten children aged 4 and 5 years old in Halabja governorate. The study lasted one year, from July 2022 to June 2023, and was ethically approved. Written informed consent was obtained from the parents or families of the children.

### Setting and data collection

A multistage cluster sampling method was employed to collect the study sample. The city’s geography was categorized into four distinct regions: north, south, east, and west. Within each of these regions, a total of 2–3 kindergartens were selected, resulting in a comprehensive selection of 10 kindergartens representing the entirety of the city. The required data for the study was collected through a structured questionnaire form from the children’s parents including the following questions: demographics of children and parents, gestational age, type of feeding during the first two years of life, complementary feeding time, educational level of parents, family size, and socio-economic status. The questionnaire was distributed among families of children in all public and private kindergartens. We implemented quality control measures and adhered to best practices, which included training our data collection team, conducting pre-tests on processes and materials, and closely monitoring data collection in the field. To determine the necessary sample size for the population, we utilized the following equation from OpenEpi (Version 3), an open-source calculator: n = [DEFF*Np (1-p)]/ [(d2/Z21-α/2*(N-1) + p*(1-p)]. According to that calculation, a sample size of 383 individuals was needed at a confidence level of 95%. However, we collected a larger sample size than this requirement to ensure greater accuracy.

### Anthropometric measurements

The height and weight of the children were measured. An electronic body scale (TCS-200-RT) was used for measuring the height. The children were taught to stand on the baseboard with their feet slightly apart. The back of the head, shoulder blades, buttocks, calves, and heels all touched the vertical board. This alignment was impossible for obese children, so they were helped to stand on the board with one or more contact points touching the board. The headboard was placed on the highest point of the head with enough strength to compress the hair. Anthropometric measurements were acquired in duplicate by the research team, with the final measurements being calculated as the mean between the two readings based on the protocols advised by WHO (10). According to the WHO Child Growth Standards and using the WHO Anthro Survey Analyser software, children were classified as “stunted” when their height-for-age z-score was below two standard deviations from the median of the same age and sex group (11).

### Eligibility criteria

Children aged 4 to 5 years old in public and private kindergartens in Halabja governorate were included. Children with skeletal deformities, genetic diseases like achondroplasia, growth hormone deficiency, and familial short stature were excluded. Additionally, children whose parents were unable to complete the questionnaire or did not complete it properly were also excluded.

### Statistical analysis

The data was collected and encoded using Microsoft Excel (2019), and then it was analyzed using the Statistical Package for Social Sciences (SPSS Version 22). Frequencies and percentages were calculated for categorical variables. The binary logistic regression test was used to compare proportions and show the possible association between stunting and the studied factors. The significant level was considered at a *p*-value < 0.05.

### Resource assessment

For assessing the available resources, all cited studies have been checked for credibility (fully peer-reviewed) (12).

## Results

### Social demographics of the children

There were 646 children in the study. Of these, 310 (48%) were boys, and 336 (52%) were girls. Most participants (87.9%) were 5 years old, while the remaining 12.1% were 4 years old. Overall, 86% were born at 9 months of gestational age, 13% were born between 7–9 months, and 1% were born at 7 months. Regarding feeding during the first 2 years of life, 35.4% of children were exclusively breastfed, 14.4% were bottle-fed, and 50.2% had mixed feeding. Also, 20% of children started complementary feeding before 6 months, while 80% started it after 6 months (Table 1).

**Table 1:** The baseline characteristics of the participants.

Variables	No. cases/frequency
Demographics	
Age	
4 years	78 (12.1%)
5 years	568 (87.9%)
Sex	
Male	310 (48.0%)
Female	336 (52.0%)
Gestational age	
9 months	556 (86.0%)
>7–<9 months	84 (13.0%)
7 months	6 (1.0%)
Type of feeding	
Breastfeeding	229 (35.4%)
Bottle feeding	93 (14.4%)
Mixed feeding	324 (50.2%)
Complementary feeding	
Before 6 months	129 (20.0%)
After 6 months	517 (80.0%)
Stunting statue	
Yes	51 (7.9%)
Male	21 (3.3%)
Female	30 (4.6%)
No	595 (92.1%)
Mothers’ age group	
15–24	20 (3.1%)
24–35	360 (55.7%)
35–45	234 (36.2%)
≥45	32 (5.0%)
Mothers’ education	
Illiterate	39 (6.0%)
Primary and Secondary school	281 (43.5%)
Diploma	167 (25.9%)
Bachelor	152 (23.5%)
Higher education	7 (1.1%)
Fathers’ education	
Illiterate	12 (1.9%)
Primary and Secondary school	341 (52.8%)
Diploma	128 (19.8%)
Bachelor	149 (23.0%)
Higher education	16 (2.5%)
Mothers’ occupation	
Housewife	436 (67.5%)
Employee	200 (31.0%)
Unknown	10 (1.5%)
Family size	
3–5 person	516 (79.9%)
6–9 person	129 (20.0%)
≥9 person	1 (0.1%)
Family socio-economic status	
Low	111 (17.2%)
Medium	373 (57.7%)
High	162 (25.1%)

No., number.

### Stunting and children-related factors

In the sample pool, the stunting prevalence was 7.9%, with 4.6% of females and 3.3% of males. Most stunted children were 5 years old (7.6%). Among stunted children, 3.6% were breastfed, 3.1% were mixed-fed, and 1.2% were bottle-fed. The majority of stunted children (6.7%) started complementary feeding after 6 months, while 1.2% started before that. Stunting affected 6.35% of term babies and 1.55% of preterm babies. When comparing age, gender, type of feeding, complementary feeding, and gestational age with stunting, no statistically significant correlation was found (*p*-value > 0.05) (Table 2).

**Table 2:** Association between nutritional status (assessed by HAZ) and gender, age groups, type of feeding, complementary feeding, and gestational age.

Variables	HAZ^*^			
	Stunting	Normal	Total	*p*-value
	No. (%)	No. (%)	No. (%)	
**Gender**				1.00
Male	21 (3.3)	289 (44.7)	310 (48)	
Female	30 (4.6)	306 (47.4)	336 (52)	
Total	51 (7.9)	595 (92.1)	646 (100)	
**Children age group**				
4 years	2 (0.3)	76 (11.8)	78 (12.1)	1.00
5 years	49 (7.6)	519 (80.3)	568 (87.9)	
Total	51 (7.9)	595 (92.1)	646 (100)	
**Type of feeding**				
Breastfeeding	23 (3.6)	206 (31.9)	229 (35.4)	0.491
Bottle feeding	8 (1.2)	85 (13.2)	93 (14.4)	
Mixed feeding	20 (3.1)	304 (47)	324 (50.2)	
Total	51 (7.9)	595 (92.1)	646 (100)	
**Complementary feeding**				
Before 6 months	8 (1.2)	121 (18.7)	129 (20)	0.715
After 6 months	43 (6.7)	474 (73.4)	517 (80)	
Total	51 (7.9)	595 (92.1)	646 (100)	
**Gestational age**				
9 months	41 (6.35)	515 (79.7)	556 (86.1)	0.266
>7–<9 months	10 (1.55)	74 (11.5)	84 (13)	
7 months	0 (0)	6 (0.9)	6 (0.9)	
Total	51 (7.9)	595 (92.1)	646 (100)	

*HAZ, height-age-Z score.

### Stunting and parent-related factors

Children whose parents completed primary and secondary school had a higher likelihood of being affected by stunting (3.7% for mothers and 3.4% for fathers), followed by those with bachelor’s degrees (2% for mothers and 2.2% for fathers) and diplomas (1.7%). However, this wasn’t statistically confirmed (*p*-value > 0.05). No significant association was found between stunting and age, occupation of mothers, family size, or family socioeconomic status (*p*-value > 0.05) (Table 3).

**Table 3:** Association between nutritional status (assessed by HAZ) and parents’ level of education, mothers’ occupation, mothers’ age, family size, and family socio-economic status.

Variables	HAZ^*^			
	Stunting	Normal	Total	*p*-value
	No. (%)	No. (%)	No. (%)	
**Mothers’ education**				1.00
Illiterate	3 (0.5)	36 (5.6)	39 (6)	
Primary and secondary school	24 (3.7)	257 (39.8)	281 (43.5)	
Diploma	11 (1.7)	156 (24.1)	167 (25.9)	
Bachelor	13 (2)	139 (21.5)	152 (23.5)	
Higher education	0 (0)	7 (1.1)	7 (1.1)	
Total	51 (7.9)	595 (92.1)	646 (100)	
**Fathers’ education**				
Illiterate	3 (0.5)	9 (1.4)	12 (1.9)	1.00
Primary and secondary school	22 (3.4)	319 (49.4)	341 (52.8)	
Diploma	11 (1.7)	117 (18.1)	128 (19.8)	
Bachelor	14 (2.2)	135 (20.9)	149 (23)	
Higher education	1 (0.1)	15 (2.3)	16 (2.5)	
Total	51 (7.9)	595 (92.1)	646 (100)	
**Mothers’ occupation**				1.00
Housewife	37 (5.7)	399 (61.8)	436 (67.5)	
Employee	14 (2.2)	186 (28.8)	200 (31)	
Unknown	0 (0)	10 (1.5)	10 (1.5)	
Total	51 (7.9)	595 (92.1)	646 (100)	
**Mothers’ age group**				1.00
15–24	1 (0.1)	19 (2.94)	20 (3.1)	
24–35	27 (4.2)	333 (51.55)	360 (55.7)	
35–45	20 (3.1)	214 (33.1)	234 (36.2)	
≥45	3 (0.5)	29 (4.5)	32 (5)	
Total	51 (7.9)	595 (92.1)	646 (100)	
**Family size**				1.00
3–5 person	37 (5.7)	479 (74.1)	516 (79.9)	
6–9 person	13 (2)	116 (18)	129 (20)	
≥9 person	1 (0.2)	0 (0)	1 (0.1)	
Total	51 (7.9)	595 (92.1)	646 (100)	
**Family socio-economic status**				1.00
Low	11 (1.7)	100 (15.5)	111 (17.2)	
Medium	27 (4.2)	346 (53.5)	373 (57.7)	
High	13 (2)	149 (23.1)	162 (25.1)	
Total	51 (7.9)	595 (92.1)	646 (100)	

^*^HAZ, height-age-Z score.

## Discussion

Stunting is an indicative factor for potential risks, as it reflects the comprehensive state of development marked by impoverished conditions, low socioeconomic status, and the incidence of chronic disorders (13). According to data compiled by the United Nations Children’s Fund (UNICEF) and WHO in 2018, approximately 149 million children under the age of 5 experienced stunting (14). This nutrition problem primarily impacts developing countries, and the highest rates of stunting in preschool children were observed in Eastern (45%), Middle (39%), and Western Africa (38%) (15).

Iraq, as a developing country, confronts challenges pertaining to economic and social development. Moreover, it contends with a multitude of environmental factors that impede the physical growth of children prior to puberty, including diseases, inadequate sanitation facilities, poor hygiene practices, limited access to adequate healthcare coverage and resources, and deficient food consumption. According to estimates from United Nations agencies, one out of eight Iraqi children die before reaching the age of five. Almost one-third of the children may suffer from malnutrition, and one-quarter are born underweight. In addition, another one-quarter lack access to safe water (3). This study was set out with the aim of assessing the risk factors and prevalence of stunting among preschool children in the Halabja governorate. The results showed that the prevalence of stunting among 646 children was 7.9%, indicating a high rate. Overall, the rate of stunting was higher than that found in Kosovo (16), yet considerably lower than in previous local studies from Baghdad (3, 8) and China (14), Nigeria (1), and Indonesia (2). The stunting prevalence in this study was similar to that of Brazil (9.9%), Vietnam (9.53%), Ethiopia (8.9%), and Iran (8.2%) (3–6).

Regarding the role of gender in stunting, there have been different outcomes (1–3, 17). Agho et al. reported that gender was a robust predictor of stunting in children aged 0–23 and 0–59 months. During infancy and childhood, girls were found to be less susceptible to stunting and severe stunting compared to boys (2). In contrast, another study revealed that, despite not being statistically confirmed, girls were more susceptible to stunting than boys, indicating that girl’s growth might be more influenced by environmental factors like infectious diseases (3). A lack of gender roles in stunting has also been reported (17). Our study supported the latter assumption.

In the literature, numerous factors associated with stunting have been discussed, including environmental factors, number of family meals per day, age, gender, socioeconomic status, parental occupation, parental education, perceived birth size, diarrhea, breastfeeding duration, complementary feeding, maternal body mass index, family size, and residency (1–3, 18–20). Akombi et al. found a significant association between the duration of breastfeeding and stunting. Children breastfed for more than 12 months were found to have a higher likelihood of experiencing stunting and severe stunting compared to those breastfed for less than 12 months. They claimed that this outcome could be attributed to various factors, including cultural influences, exclusive breastfeeding practices, socioeconomic conditions, the time of initiating complementary feeding, the quality of complementary foods, and the educational status of the mothers (1). In the present study, there was no statistically significant association between stunting and age. The highest percentage of stunted children were breastfed children (3.3%). Stunting is a reflection of chronic malnutrition, so this finding may be explained by improper nutrition of the mother during pregnancy and the lactation period. Additionally, the high percentage of stunting in those who started complementary feeding after 6 months may be due to the malpractice of giving food, as the family thought it was time to give everything to their child, but this may be a misconception, consequently leading to stunting. Moreover, this study showed that children born at full term had a higher percentage of stunting than those born prematurely; this is in contrast to the study of Sullivan et al. (21). The explanation for this in our study may be that families of preterm babies took care of their babies more seriously than those of full-term babies and more carefully followed the pediatrician’s advice because of the babies’ condition.

It has also been found that infants who were perceived by their mothers as being of smaller or average size at birth displayed a greater propensity for experiencing stunted and severely stunted growth in comparison to larger newborns. This diminished birth size may be attributed to inadequate maternal nutrition during the gestational period. Throughout this critical period, the child is entirely reliant on the mother for nourishment through the placenta. Consequently, any deprivation of nutrition by the mother can have detrimental effects on the child’s growth and optimal development (1). Furthermore, the study presented a significant association between the mother’s BMI and stunting in children under 5 years old. Mothers with a BMI below 18.5 kg/m^2^ were more prone to have stunted children when compared to mothers with a BMI of 25 kg/m^2^ or higher (1). Unfortunately, in this study, we could not represent any data regarding that, but another reason for such a higher incidence in full-term babies might be due to the dual mentioned factors.

Akombi et al., in a study on the Nigerian population, found that children from economically disadvantaged households faced a higher risk of stunting compared to children from more affluent households. This phenomenon could be attributed to the limited financial resources available to poorer families, resulting in inadequate spending on proper nutrition and making them more vulnerable to infectious diseases due to not reaching basic health care services (1). Comparable findings were derived from cross-sectional studies conducted in Iran (7) and Nepal (18). However, Sarma et al. demonstrated that children of rich families may also be affected by stunting (20).

Offspring of educated fathers have shown a reduced likelihood of experiencing stunting in comparison to children born to fathers with limited education. Moreover, children born to educated mothers who were breastfed demonstrated a decreased probability of severe stunting in contrast to children born to uneducated mothers who were not breastfed. These findings underscore a positive correlation between breastfeeding and parental education in fostering the development of their children (1, 18, 22). Another study showed that children whose mothers had bachelor’s degrees were less likely to be affected by stunting compared to those whose mothers had diplomas or lower educational attainment. They explained that mothers with higher levels of education had greater knowledge about their children’s health and nutrition, leading to improved childcare practices, increased utilization of health services, and better attention to hygiene and sanitation (7). Despite all of that, other studies have found that fathers’ education was not correlated with being affected by stunting or not (7, 23). In addition, it has been reported that children with educated mothers showed a higher likelihood of being stunted than those whose mothers had no formal education. This was explained by the possibility that educated mothers were more likely to be employed, which might have led to reduced time available for childcare responsibilities (5). Giao et al. also revealed that housewife mothers and mothers with seller occupations were less likely to have stunted children than mothers employed in the public sector (6). Regarding the relationship between stunting and family education, this study showed a high prevalence of stunting in children whose mothers completed primary and secondary school, followed by those with bachelor’s degrees and diplomas. These results may be explained by the fact that illiterate mothers in our community easily follow the instructions and advice given by experts in the field compared to those who have access to getting steps by themselves. In our study, the father’s education played a reverse role in comparison to the previously mentioned studies (1, 18, 22). This may be related to the fact that families with illiterate fathers still have traditional feeding practices and easily follow instructions.

Our findings showed that a high percentage of the mothers of stunted children were housewives, although this may be due to the fact that most mothers in our region were housewives or families with employed mothers were economically more stable. The mothers of the stunted children were mostly young, similar to the study by Giao et al. (6). Children from middle-income families had a higher incidence than those from high- and low-income families. This finding might be due to the inaccuracy of the information given about family income in our sample data.

The major limitations of this study can be attributed to the nature of the study design, which is subjected to bias by respondents providing inaccurate data, and the collection of data in a small geographical area. However, this study can be a starting point for further studies with larger samples occupying a wider geographical area to accurately point out the factors and prevalence of the disease in the Iraqi population. Research to examine whether the historical chemical warfare has enduring effects on growth in both recent and future generations could be intriguing.

## Conclusion

The prevalence of stunting in the studied population was 7.9%. However, we could not find any significant association between the studied factors and stunting. Thus, the factors that may significantly affect stunting in our area of study, especially the historical chemical warfare side effects, need to be more extensively investigated in future studies.

## Data availability statement

The datasets presented in this article are not readily available because if needed, after that, it will be uploaded. Requests to access the datasets should be directed to fahmi.hussein@univsul.edu.iq.

## Ethics statement

The study involved humans and was approved by the University of Halabja, Halabja, Kurdistan, Iraq. The study was conducted in accordance with the local legislation and institutional requirements. Written informed consent for participation in this study was provided by the participants’ parents.

## Author contributions

HM: Data curation, Investigation, Writing – review & editing. ZN: Conceptualization, Formal analysis, Writing – original draft. KHH: Methodology, Validation, Visualization, Writing – review & editing. DH: Data curation, Investigation, Validation, Writing – review & editing. HA: Conceptualization, Validation, Visualization, Writing – original draft, Writing – review & editing. KMH: Conceptualization, Data curation, Validation, Writing – review & editing. HK: Data curation, Investigation, Methodology, Writing – review & editing. BM: Conceptualization, Methodology, Validation, Visualization, Writing – review & editing. FF: Writing – review & editing. BA: Data curation, Methodology, Supervision, Validation, Writing – review & editing. FK: Supervision, Writing – review & editing. SM: Data curation, Formal analysis, Validation, Visualization, Writing – review & editing.

## Footnotes

^1^ https://krso.gov.krd/en/indicator
